# Supplementary figures and images for: CLOCK and TIMELESS regulate rhythmic occupancy of the BRAHMA chromatin-remodeling protein at clock gene promoters
Source: PLoS Genet. 2023 Feb 21;19(2):e1010649. doi: 10.1371/journal.pgen.1010649 (PMC9983840; doi:10.1371/journal.pgen.1010649)

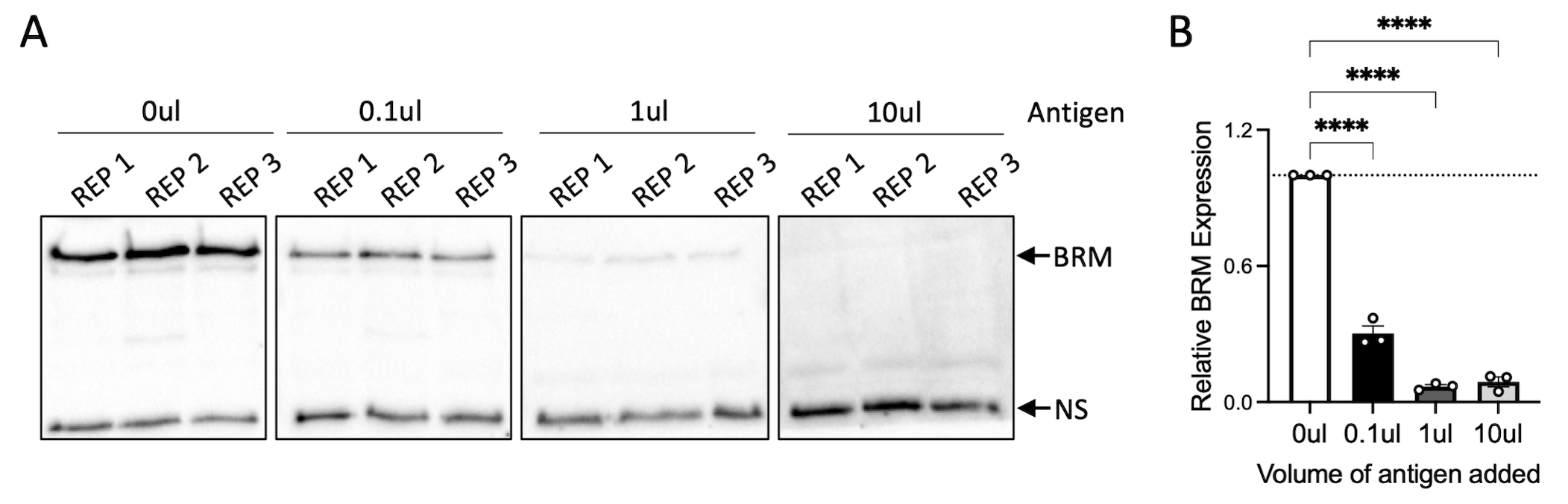

Supplement: S1 Fig — (A) The BRM antibody was incubated with a dilution series of the BRM antigen (0.1ul, 1ul, and 10ul at 1ug/ul) prior to detecting BRM in protein lysate extracted from w1118 flies collected at ZT16. The non-specific band is denoted as NS. (B) BRM signal was normalized to the NS signal (n = 3). Each data point represents a biological replicate. Error bars represent ±SEM. Asterisks denote significant p-values: ****p<0.0001. (TIFF) [file pgen.1010649.s001.tiff]

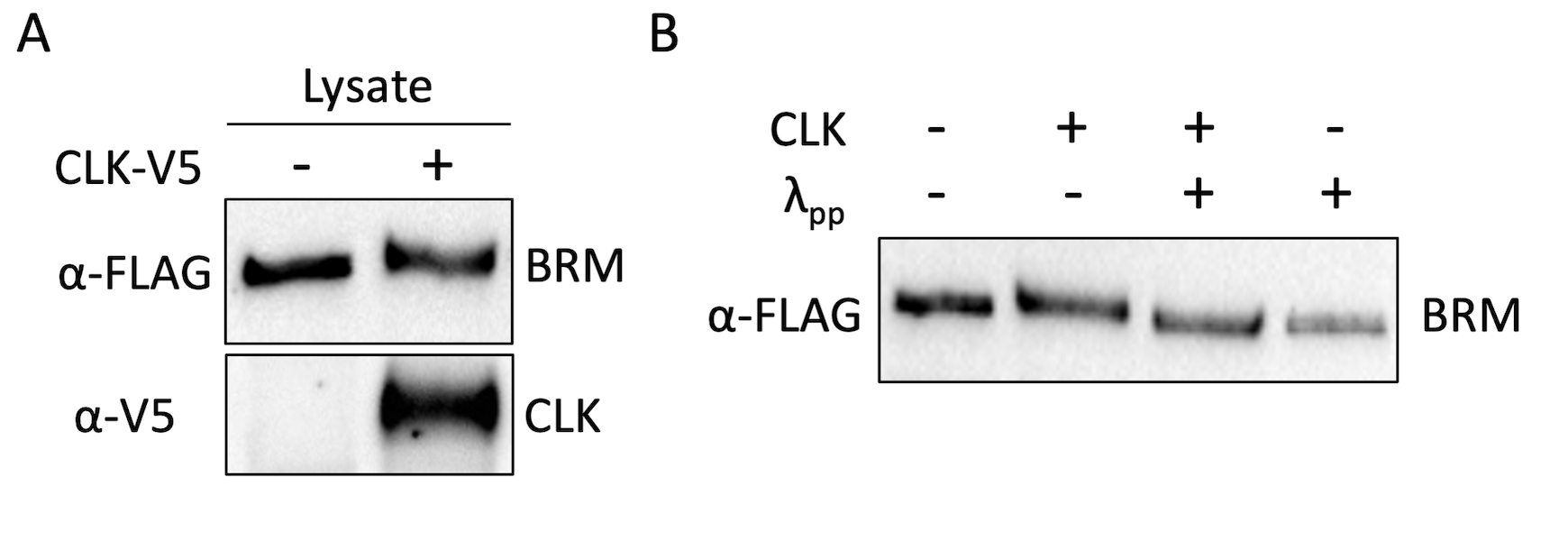

Supplement: S2 Fig — (A) BRM (top panel) and CLK (bottom panel) expression prior to lambda phosphatase (λpp) treatment in protein lysate from S2 cells expressing either BRM alone or BRM co-expressed with CLK. (B) BRM protein after treatment with λpp. (TIFF) [file pgen.1010649.s002.tiff]

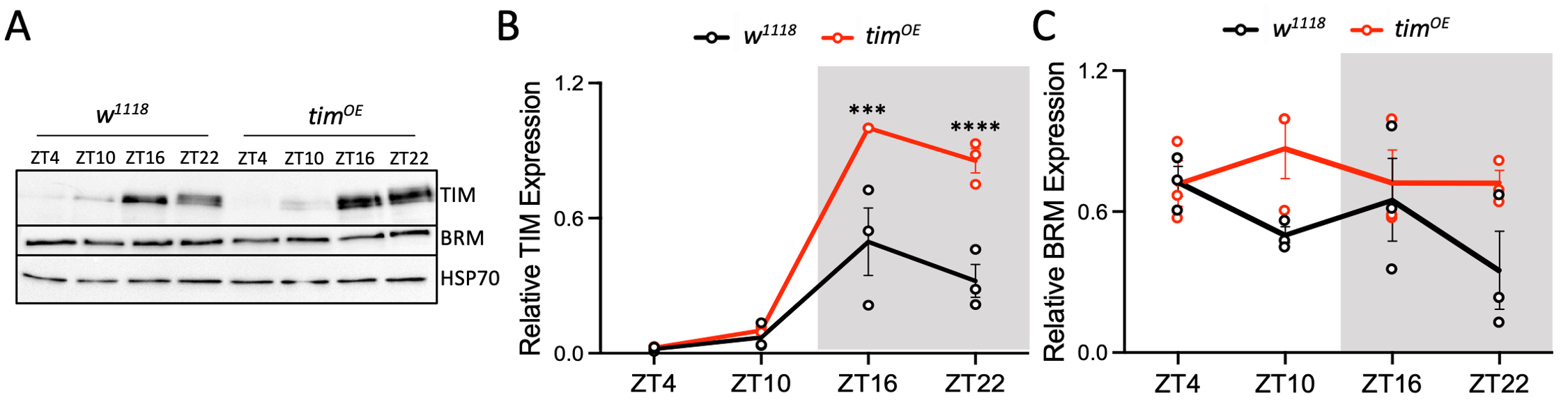

Supplement: S3 Fig — (A) TIM (top panel) and BRM (middle panel) protein in w1118 and w1118;ptim(WT) fly heads collected at the indicated time points on LD3. w1118;ptim(WT) flies are denoted as timOE flies. HSP70 (bottom panel) was used as a loading control. (B-C) Normalized (B) TIM and (C) BRM expression in w1118 (black) and timOE (red) flies (n = 3). Each data point represents a biological replicate. Error bars represent ±SEM. The grey background denotes the dark phase of the LD cycle. (TIFF) [file pgen.1010649.s003.tiff]

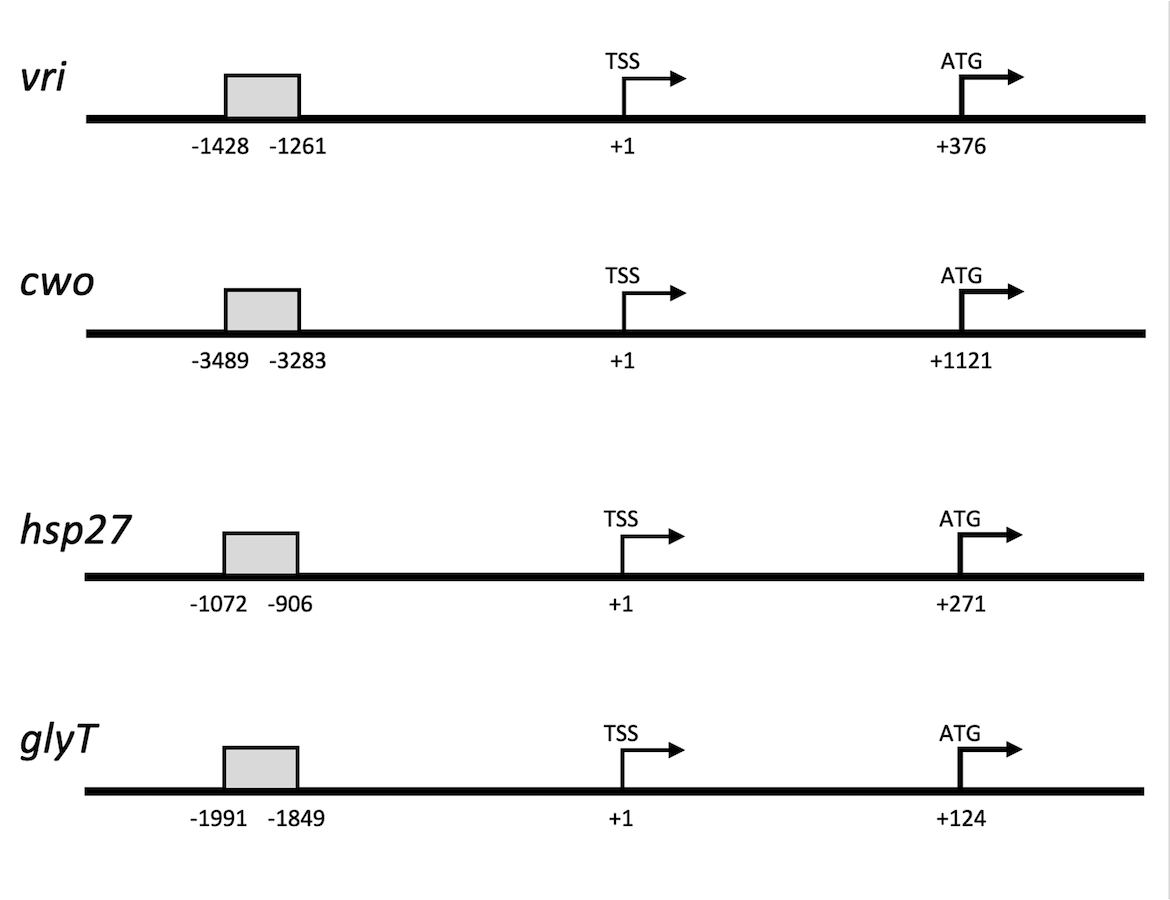

Supplement: S4 Fig — Schematic of region amplified by primers (grey) used in ChIP to assess BRM occupancy at the promoters of vrille (vri), clockwork orange (cwo), heat shock protein 27 (hsp27), and glycine transporter (glyT). Positions are relative to the transcription start site (TSS). Locations of other ChIP primers are shown in [37]. (TIFF) [file pgen.1010649.s004.tiff]
