# Supplementary material for: CLOCK and TIMELESS regulate rhythmic occupancy of the BRAHMA chromatin-remodeling protein at clock gene promoters
Source: PLoS Genet. 2023 Feb 21;19(2):e1010649. doi: 10.1371/journal.pgen.1010649 (PMC9983840; doi:10.1371/journal.pgen.1010649)
Supplement: S1 Table — (DOCX) [file pgen.1010649.s005.docx]

**S1 Table: Sequences for primers used for generation of BRM antigen, Chromatin Immunoprecipitation-qPCR, and steady-state mRNA analysis.**

| **Purpose** | **Primer Name** | | **5'-Sequence-3'** | **Reference** |
| --- | --- | --- | --- | --- |
| Antigen generation | *brm* (3961) Forward | | GTG TGG ATC CGA TGA GTC CGA GCT ACC CGA CTG G | This study |
|  | *brm* (4518) Reverse | | GTG TGC GGC CGC CTA AGA CGC CTC TTC GTT GTA GAT CTG | This study |
| Chromatin Immunoprecipitation | *per* CRS Forward | | TGC CAG TGC CAG TGC GAG TTC G | Kwok et al. 2015 [1] |
|  | *per* CRS Reverse | | TGC CTG GTG GGC GGC TGG | Kwok et al. 2015 [1] |
| Chromatin Immunoprecipitation | *tim E-box 1* Forward | | ACG TTG TGA TTA CAC GTG AGC CG | Kwok et al. 2015 [1] |
|  | *tim E-box 1* Reverse | | TAC ACA CAC TGA CCG AAA CAC CC | Kwok et al. 2015 [1] |
| Chromatin Immunoprecipitation | *vri* (-1428) Forward | | AAC CAG ACA GTT TGG TGG CTG GG | This study |
|  | *vri* (-1261) Reverse | | CAG TGC TAG CTA ACT ATT TGA ACT CGT C | This study |
| Chromatin Immunoprecipitation | *cwo* (-3283) Forward | | TTC TCC GGC AGT TGC ACC G | This study |
|  | *cwo* (-3489) Reverse | | TTA CTC ATG TGC CAC ATT CTC G | This study |
| Chromatin Immunoprecipitation | FBgn0003638 Forward | | ACT GCG TAT TCA GGA TAC ATG CC | Kwok et al. 2015 [1] |
|  | FBgn0003638 Reverse | | TGT CCA CTT TAA TTG ATT GCG TGG | Kwok et al. 2015 [1] |
| Chromatin Immunoprecipitation | *hsp27* (-1027) Forward | | TGA ATG TAA GGA ACT TCA GTC AAG G | This study |
|  | *hsp27* (-906) Reverse | | TGC TAT AAG GAC GTA CAT AAC GTA C | This study |
| Chromatin Immunoprecipitation | *glyT* (-1991) Forward | | TGA GCA GCG ATC GAC GCT GAC G | This study |
|  | *glyT* (-1849) Reverse | | TCG GTG CTG CAA AGC GCC GTT C | This study |
| mRNA analysis | *brm b3* qPCR Forward | | AGC CAG GTA CAA GCT GAA C | Kwok et al. 2015 [1] |
|  | *brm b3* qPCR Reverse | | TGA TCA TTT CGT CAT CGG | Kwok et al. 2015 [1] |
| mRNA analysis | *cbp20* qPCR Forward | | GTC TGA TTC GTG TGG ACT GG | Majercak et al. 2004 [2] |
|  | *cbp20* qPCR Reverse | | CAA CAG TTT GCC ATA ACC CC | Majercak et al. 2004 [2] |
| mRNA analysis | *per* qPCR Forward | | GAC CGA ATC CCT GCT CAA | Kwok et al. 2015 [1] |
|  | *per* qPCR Reverse | | GTG TCA TTG GCG GAC TTC | Kwok et al. 2015 [1] |
|  | |  |  |  |
|  |  |  |  |  |

**References**

1. Kwok RS, Li YH, Lei AJ, Edery I, Chiu JC. The Catalytic and Non-catalytic Functions of the Brahma Chromatin-Remodeling Protein Collaborate to Fine-Tune Circadian Transcription in *Drosophila*. Emery P, editor. PLOS Genet. 2015;11: e1005307. doi:10.1371/journal.pgen.1005307

2. Majercak J, Chen W-F, Edery I. Splicing of the period Gene 3′-Terminal Intron Is Regulated by Light, Circadian Clock Factors, and Phospholipase C. Mol Cell Biol. 2004;24: 3359–3372. doi:10.1128/MCB.24.8.3359-3372.2004
